# Different roles for different bones**—**Location matters for blood production

**DOI:** 10.1002/hem3.127

**Published:** 2024-07-19

**Authors:** David G. Kent

**Affiliations:** ^1^ Department of Biology, Centre for Blood Research, York Biomedical Research Institute University of York York UK

Laboratories studying hematopoietic stem cell (HSC) biology from across the world all have their own way of doing things, and this extends not just to the various cell surface marker combinations used to isolate HSCs but also to the more mundane aspects of cell isolation and preparation. At the very beginning of an HSC experiment, all researchers are faced with the question of which bones to obtain their bone marrow sample from. In the mouse, where options are more numerous, researchers commonly obtain marrow samples from the tibia, femur, hip, sternum, or spine. Some researchers then proceed to isolate the marrow by flushing, others by centrifuging bones, and still others by crushing. Yet, the vast majority of studies simply report that mouse bone marrow was isolated, and where it is specified, it is rarely looked at as a major experimental variable (e.g., a study with bone marrow from the tibia and femur is not viewed differently to one that also obtains marrow from spine and hip). Does it really matter which bone (or which part of which bone) cells are isolated from? Recent evidence suggests that the answer is a resounding “yes.”

While numerous anecdotes have circulated around various conference circuits and between highly specialized HSC labs, it is rare to see published studies that go into the fine detail of differences in the anatomical location of bone marrow blood cells. That said, studies have been cropping up in various guises over the years, and a major landmark study came out this year from Daniel Lucas' group^1^ that added fuel to the fire and appears to cement the unique roles of different bones in blood cell production. One of the earliest studies was from Brian Lord in the 1970s where colony‐forming cells were isolated from either the central marrow shaft (dubbed “axial”) or the edges of the bone (named “marginal”) and distinct numbers and types of colonies resided in each cell preparation.^2^ This study preceded a large number of studies that focused on different types of bone and bone matrix, different proposed HSC niches, and different neighboring cell types (reviewed in Comazzetto et al.^3^).

Another provocative study emerged from David Bryder's lab in 2015^4^ that challenged the notion of transplantable HSCs being equally distributed across the skeleton of a repopulated mouse. Following 16 weeks of transplantation and monitoring of overall chimerism in the blood, each of the right and left legs (tibia, femur, and hip) were assessed for donor chimerism, and the differences between bones were substantial, with some animals showing chimerism nearly exclusively in a single bone. Experimentally, this again challenges researchers to not consider that all marrow is equal irrespective of location.

Coming back to the Lucas lab study and why it has made such a big impact in the field, they undertook a skeleton‐wide imaging study to define the context of different stem and progenitor cells with respect to location and cell neighbors while also conducting a test of induced stress to see if all bones behave similarly to systemic stress. Prior to stress and at all skeletal locations observed, Wu et al. showed that HSCs and a range of multipotent progenitors were present throughout the marrow and were particularly enriched near megakaryocytes and that lineage‐restricted progenitors were subsequently recruited into vessels. Following a variety of stresses (hemorrhage, infection, and granulocyte colony‐stimulating factor (GCSF) treatment), this basic structure was maintained. What emerged as different following stress induction, however, was the committed progenitor activity in these different anatomical locations. The tibia responded to GCSF by nearly doubling the number of granulocyte progenitors and mature neutrophils, whereas the sternum from the same mice had reduced numbers of granulocyte progenitors and mature neutrophils. Together, these data showed that, depending on the anatomical location, stress hematopoiesis induced completely different responses. While the authors did not go into much detail as to what the biological reasoning for this particular response might be, the technology of lineage tracing and whole‐mount tissue monitoring permits these questions to be asked for the first time.

Overall, these studies represent a selection of data that challenge the idea that all bone marrow can be treated equivalently. At a minimum it should prompt researchers to accurately report on the origins of their bone marrow samples and the locations of their sampling in post‐transplantation or post‐stimulation studies. Moving beyond simply reporting, though, it is clear that there is fascinating biology to be unraveled if we take anatomical location into account, and it is exciting to see such enabling technologies emerging—the next challenge will be linking these observations to function.^5^

## AUTHOR CONTRIBUTION

David G. Kent is the sole contributor to this article.

## CONFLICT OF INTEREST STATEMENT

The authors declare no conflict of interest.

## FUNDING

Work in the D.G.K. laboratory is supported by the Bill and Melinda Gates Foundation (INV002189), an ERC Starting Grant (ERC‐2016‐STG‐715371), a Cancer Research UK Programme Foundation Award (DCRPGF\100008), and the Medical Research Council (MC_PC_21043).

## Data Availability

Data sharing is not applicable to this article as no new data were created or analyzed in this study.
